# Site-Specific Transient Receptor Potential Channel Mechanisms and Their Characteristics for Targeted Chronic Itch Treatment

**DOI:** 10.3390/biom14010107

**Published:** 2024-01-15

**Authors:** Eun Jin Go, Ji Yeon Lee, Yong Ho Kim, Chul-Kyu Park

**Affiliations:** 1 Gachon Pain Center and Department of Physiology, College of Medicine, Gachon University, Incheon 21999, Republic of Korea; navy2474@gachon.ac.kr; 2 Department of Anesthesiology and Pain Medicine, Gil Medical Center, Gachon University, Incheon 21565, Republic of Korea; easy95@gilhospital.com

**Keywords:** chronic itch, TRP channels, skin, sensory neurons

## Abstract

Chronic itch is a debilitating condition with limited treatment options, severely affecting quality of life. The identification of pruriceptors has sparked a growing interest in the therapeutic potential of TRP channels in the context of itch. In this regard, we provided a comprehensive overview of the site-specific expression of TRP channels and their associated functions in response to a range of pruritogens. Although several potent antipruritic compounds that target specific TRP channels have been developed and have demonstrated efficacy in various chronic itch conditions through experimental means, a more thorough understanding of the potential for adverse effects or interactions with other TRP channels or GPCRs is necessary to develop novel and selective therapeutics that target TRP channels for treating chronic itch. This review focuses on the mechanism of itch associated with TRP channels at specific sites, from the skin to the sensory neuron, with the aim of suggesting specific therapeutic targets for treating this condition.

## 1. Introduction

Itch, or pruritus, is defined as an unpleasant sensation that elicits an innate scratching response. Irritants such as insect bites and contact with poisonous plants, leading to acute itch, can be readily alleviated by scratching. This response may be natural; however, it can become pathological if immense suffering from chronic itch lasts more than 6 weeks [1]. Chronic itch is classified into four categories: dermatologic, systemic, neuropathic, and psychogenic [2,3,4]. Dermatologic itch conditions in atopic dermatitis (AD), psoriasis, and xerosis stem from skin diseases [3]. Systemic itch arises from organs other than the skin and always accompanies diseases of organs, such as conditions in cholestatic pruritus and uremic pruritus [5]. Neuropathic itch results from nerve injury and arises from diseases of the central or peripheral nervous system [6]. Examples of neuropathic itch include neuropathy, nerve compression or irritation, multiple sclerosis, and brain tumors. Psychogenic itch is caused by psychological or psychiatric disorders [7]. Though chronic itch has been significantly impairing the quality of patients’ lives by interrupting their sleep or causing anxiety or depression [8,9], an understanding of the molecular and neural mechanisms of chronic itch remains limited.

Itch has been regarded as a sub-modality or a mild form of pain [10] due to the similarities in the two sensations [11]. In 2009, Chen et al. discovered itch-specific neurons in mice and claimed that itch and pain are two distinct sensations [12,13]. Though not identical, the two sensations are closely related, as itch-sensing nerve fibers express two families of receptors: G protein-coupled receptors (GPCRs) and the transient receptor potential (TRP) [14,15]. TRP channels are non-selective ion channels primarily located on the plasma membrane in various cells and are divided into six families: TRPV (vanilloid), TRPA (ankyrin), TRPM (melastatin), TRPP (polycystin), TRPC (canonical), and TRPML (mucolipin) [16]. Among the members of the TRP channel family, TRPV1, TRPA1, TRPV3, and TRPV4 are especially known to be involved in itch transduction [17,18,19].

In this review, we provide an overview of the current understanding of the mechanisms of itch, beginning with the initiation of itch at the site of the skin and tracing its progression to the itch sensory neurons where the sensation is perceived and conveyed. Additionally, we discuss the role of TRP channels as key players in chronic itch, aiming to suggest specific therapeutic targets for treating this condition.

## 2. Mechanism of Itch from Skin to Peripheral Sensory Neuron

During an episode of acute itch, the skin is distributed by a breach or chemical insult (such as chemical mediators or insect bites), which in turn follows a pruriceptive itch [20]. Free nerve endings of peripheral sensory nerve fibers terminate in the skin and detect the changes in the local chemical environment. These changes involve various chemical mediators, including histamine, serotonin, proteases, chemokines, and cytokines released by keratinocytes and local immune cells [21,22].

The peripheral sensory nerve fibers conveying the itch sensation are broadly classified into unmyelinated C-fibers and lightly myelinated Aδ-fibers [23,24]. Unmyelinated C-fibers detect and transmit pruriceptive information more slowly than myelinated Aδ-fibers. Unmyelinated C-fibers are subdivided into peptidergic and non-peptidergic fibers [24,25,26]. Peptidergic C-fibers contain large vesicles that release inflammatory neuropeptides such as substance P (SP) and calcitonin gene-related peptide (CGRP) at both central and peripheral terminals, whereas non-peptidergic fibers express the purinergic receptor P2 X3 and isolectin B4 (IB4) [26]. Ringkamp et al. reported the involvement of A fibers in itch perception, demonstrating attenuated itch sensation when the conduction of myelinated fibers was selectively blocked [23]. Among many subtypes of primary afferent nerves originating from the distal, a single nerve subtype consisting of an itch receptor is responsible for transducing pruriceptive stimuli to primary afferent dorsal root ganglion (DRG) neurons and then to the higher centers [27]. In the itch signal transduction, the TRP family is recognized as a key player. Among the TRP family in mammals, the five subgroups, the TRP vanilloids 1 (TRPV1), TRPV3, TRPV4, TRPA 1, and TRPM 8, are predominantly implicated in itch transduction [28].

One of the most commonly studied pruritogen, contributing to the initiation and modulation of itch sensation, is histamine, secreted by various cells, including T-cells, mast cells, and keratinocytes [29]. Based on this mechanism, itch is generally classified as either histamine-dependent or histamine-independent [30]. Acute itch, manifested as changes in the local chemical environment, can be detected through histamine-mediated mechanisms; however, chronic itch typically involves histamine-independent mechanisms [14]. Understanding how pruritogens interact with specific TRP channels provides insights into the molecular mechanisms of itch transduction. Furthermore, targeting these interactions holds potential for developing therapeutic interventions to alleviate itching associated with various skin conditions.

### 2.1. Detection of Itch through the Skin

When the skin barrier is damaged due to genetic, inflammatory, and environmental causes, passive water loss through the skin increases and induces the sensation of itch through non-myelinated C-fiber activation [31]. The itch–scratch cycle initiation leads to epidermal damage, perpetuating the itch sensation [31]. Various itch-sensing receptors are characterized in the skin and immune cells. Histamine receptors H1R, H2R, H3R, and H4R are distributed across various tissues [32]. H1R binds to G_q_/G_11_ proteins and activates phospholipase A2 [33], phospholipase Cβ3 (PLCβ3) [34], protein kinase Cδ (PCKδ) [35], and TRPV1 [35,36], resulting in calcium influx and firing action potentials in primary sensory neurons. Mas-related G protein-coupled receptors (Mrgprs) are broadly required for detecting both exogenous pruritogens and endogenous itch mediators from keratinocytes and immune cells [37]. Serotonin (5-hydroxytryptamine, 5-HT) receptors (HTRs) are expressed in the skin by immune cells and sensory neurons, influencing immune response and sensory perception [38]. In mice, 5-HT is released by mast cells, acting as a pruritogen at lower doses than histamine [39]. Toll-like receptors (TLRs) are expressed across keratinocytes, immune cells, and neurons, and they detect pathogen-associated molecular patterns [40]. Among TLRs, TLR3, TLR4, TLR5, and TLR7 have been known to mediate itch in mice [41,42]. Protease-activated receptors (PARs), especially PAR2 and PAR4, have been implicated in itch initiation by detecting various exogenous and endogenous proteases [43,44,45]. They are expressed by keratinocytes, immune cells, and neurons [46]. Finally, cytokines-related type 2 immune responses are increasingly recognized for their role in itch perception. Keratinocytes release TSLP, a major instigator of the T helper (Th) 2 response, in response to various stimuli, including allergy and proteolytic PAR2 activation [47,48,49]. High TSLP expression in the skin is reported as a feature of atopic dermatitis (AD) [50].

### 2.2. Transduction of the Itch Signal through the Peripheral Sensory Neuron

Two theories for pruriceptive sensory processing in the nervous system have been postulated: the specificity and pattern theories [51,52,53]. The former asserts the existence of specific types of sensory nerve fibers and spinal cord neurons that transmit itch-specific information to the central nervous system; however, the latter states that the itch sensation is encoded by many sensory receptors and spinal cord neurons that compose the collective pattern of neuronal activity, determining the ultimate sensation experienced. Cumulative evidence increasingly supports the specificity theory over the pattern theory, although much remains to be learned.

As multiple nerve subtypes are activated, the specific subtypes of sensory neurons transducing pruriceptive itch become complex. For example, cutaneous C-fibers respond to both histamine and capsaicin [54,55]. Similar to C-fibers, an evaluation of the sensory nerves that express MrgprA3-expressing DRG neurons also express receptors for histamine, gastrin-releasing peptide (GRP) [56], and capsaicin (TRPV1) [57]. Han et al. found that the ablation of MrgprA3-expressing neurons in the DRG reduced itch behavior without disturbing pain [17]. Moreover, the expression of TLR7 in primary sensory neurons is required specifically for inducing itch, excluding pain. [41]. However, given the limitations of these studies, the potential role of the investigated molecules or neurons in mediating pain cannot be dismissed [14]. Pruriceptors on free nerve endings in cutaneous primary sensory neurons are activated by pruritogens and evoke an itch sensation [58]. Besides pruritogens, various inflammatory mediators, including adenosine 5′-triphosphate (ATP), thymic stromal lymphopoietin, endothelins, prostaglandins, nitric oxide, histamine, and serotonin, are released by keratinocytes and directly sensitize or activate primary sensory neurons to initiate itch signal [59]. Similarly, pruritogenic inflammatory mediators are also released by innate immune cells, including mast cells, macrophages, neutrophils, and dendritic cells [60], and the interplay between adaptive immune cells and neurons then plays a crucial role in initiating the itch signal [61,62,63].

## 3. Functional Roles of TRP Channels in the Skin

**Figure 1:**
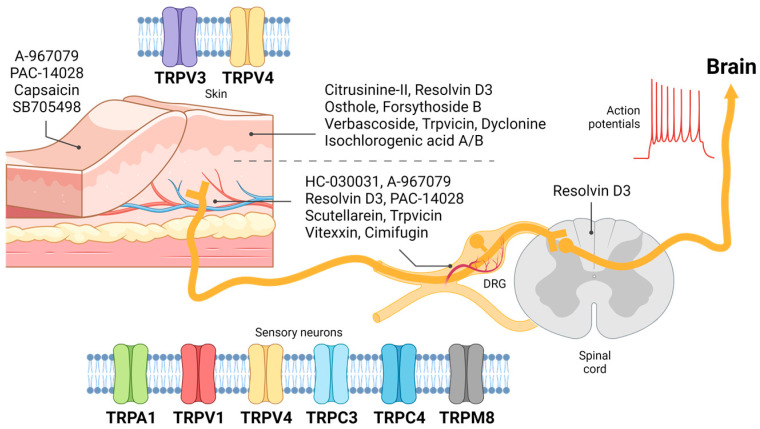
Expression of transient receptor potential (TRP) receptors in skin keratinocytes and sensory neurons concerning pharmaceutical administration routes. The expression of TRPV3 and TRPV4 has been observed in the skin, where the efficacy of TRPA1 antagonist A-967079, TRPV1 antagonist PAC-14028 and SB705498, and TRPV1 agonist capsaicin has been examined. TRPV3 antagonist citrusinine-II, osthole, forsythoside B, verbascoside, isochlorogenic acid A and B, dyclonine, trpvicin, and TRPV1 antagonist resolvin D3 are locally administered via intradermal or transdermal injection. Sensory neurons express TRPA1, TRPV1, TRPV4, TRPC3, TRPC4, and TRPM8. Systemic deliveries of TRPA1 antagonists HC-030031 and A-967079, TRPV1 antagonists resolvin D3 and PAC-14028, TRPV3 antagonists scutellarein and trpvicin, and TRPV4 antagonists vitexin and cimifugin have been investigated for their potential antipruritic effects. These compounds modulate the transmission of itch signals to the brain.

TRP channels are expressed in various excitable and unexcitable cells [64,65,66,67,68,69]. Studies have also revealed that TRP channels are involved in regulating skin physiology [70,71,72,73,74,75]. Despite the limited studies on TRP channels associated with skin and epidermis in itch, TRPV3 and TRPV4 channels (Figure 1) have been studied.

### 3.1. Skin TRPV3 in Itch

TRPV3 is a warm temperature (>33 °C)-sensitive non-selective cation channel expressed in skin keratinocytes [73,76,77,78] and poorly detected in DRG neurons or the spinal cord [73]. Similar to other polymodal TRP channels, TRPV3 is also activated by chemicals such as eugenol, carvacrol, thymol, camphor, and 2-APB [76,79]. Notably, TRPV3 is expressed in keratinocytes in mice and not in the peripheral or central nervous system, like DRG neurons or the spinal cord [73]. Studies using TRPV3 knockout mice showed markedly diminished heat responses, whereas other sensory stimuli were maintained [76]. Moreover, hair abnormalities were also observed in TRPV3 knockout mice [76,80]. Therefore, the physiological role of TRPV3 for thermosensation, hair regulation, and dermatitis has been highlighted.

In the context of itch, in vivo studies using gain-of-function TRPV3 mutation (Gly573 to Ser or Cys) in DS-Nh mice showed hairless and AD-like symptoms with spontaneous scratching [80,81]. AD is an inflammatory skin disease with intractable, chronic itch [82,83]. Moreover, gain-of-function TRPV3 Gly573Ser mutant in mice keratinocytes led to similarities with the clinical symptoms of human AD, such as skin inflammation, pruritus, immune cell infiltration, hyperkeratosis, upregulation of nerve growth factors, and systemic symptoms with elevated proinflammatory cytokines [84]. While TRPV3 Gly573 mutation mice indicated the involvement of TRPV3 in itch, the Gly573 missense mutation was also discovered in Olmsted syndrome, which is a rare congenital disease characterized by skin hyperplasia, diffuse palmoplantar keratoderma, alopecia, and severe pruritus [85]. TRPV3 knockout mice showed skin-related loss-of-function phenotypes, including curly whiskers, wavy hair, a thin stratum corneum, and misaligned hair follicles; however, they did not show itch-related scratching behaviors [86]. Moreover, TRPV3 lacking keratinocytes impaired protease-activated receptor 2 (PAR2) function in response to PAR2 agonists, leading to reduced neuronal activation and scratching behaviors [87]. Overall, these studies suggest a major role of TRPV3 in pruriceptive itch through skin keratinocytes.

### 3.2. Skin TRPV4 in Itch

TRPV4 is another temperature (27 and 35 °C)-activated channel [88], mainly expressed in keratinocytes, although less than TRPV3 in keratinocytes and in much higher levels than those found in DRG neurons [89]. TRPV4 is involved in skin barrier recovery [90], intercellular junction formation in keratinocytes [91], and intracellular calcium concentration; thus, accelerating barrier recovery after stratum corneum disruption [92,93].

A recent study on human chronic pruritus found increased expression in epidermal TRPV4 [94], suggesting a crucial role of TRPV4 in pruritus. Several TRPV4 knockout studies showed the role of TRPV4 in 5-HT- or histamine-induced itch. Reduced scratching behaviors in response to 5-HT were displayed in TRPV4 knockout mice, compared to wild-type mice [95]. In global and keratinocyte-specific TRPV4 knockout mice, scratching responses induced by histaminergic pruritogens, such as histamine, compound 48/80, and ET-1, were significantly reduced [96,97], suggesting an important role of TRPV4 in histamine-induced itch. Although controversial, the number of non-histaminergic pruritogen chloroquine (CQ)-induced scratching behaviors was increased in global TRPV4 knockout mice [95]. On the contrary, while one study found that both global or skin-specific TRPV4 knockout mice showed no effect of CQ-induced scratching [97], another study showed a significant attenuation in CQ-induced scratching behaviors in global TRPV4 knockout mice [96]. Recently, lysophosphatidylcholine (LPC), a cholestatic pruritogen, was found to directly activate TRPV4 in skin keratinocytes, triggering micro-RNA-146a release to activate TRPV1 pruriceptor neurons [98]. This study proposed the critical role of skin as a sensory organ with a new pathway of pruriception. Furthermore, the involvement of TRPV4 is supported by the deletion of TRPV4 in macrophages and keratinocytes, which showed reductions in both allergic and nonallergic chronic itch in mice [99,100]. Thus, the evidence represents TRPV4 as a potential therapeutic target for both allergic and nonallergic chronic itch conditions.

## 4. Functional Roles of TRP Channel in Sensory Neurons

Before the signal transduction of itch from the periphery in the skin to the central nervous system (i.e., spinal cord and brain), the signals pass through the DRG sensory neurons. Various TRP channels in sensory neurons are activated by external and internal itch mediators and metabolic byproducts via GPCRs, TLRs, integrin receptors, and immune receptors complex [101], and the sensory neurons are depolarized to transmit signals. Among the TRP channels, TRPA1, TRPV1, TRPV4, TRPM8, TRPC3, and TRPC4 (Figure 1) are generally known for their itch generation and transduction from sensory nerves such as DRG neurons.

### 4.1. Sensory TRPA1 in Itch

TRPA1 is a non-selective cation channel named after cytosolic N-termini with 14 ankyrin repeats and is activated by noxious cold temperature, mechanical sensation, electrophilic compounds (allyl isothiocyanate, cinnamaldehyde, diallyl disulfide, and allicin) as well as endogenous reactive oxygen species (hydrogen peroxide and 4-hydroxynonenal) [102]. Besides the physiological role of sensory detection through thermal and chemical stimuli, TRPA1 also contributes to the chronic [103] and acute histamine-independent pruritis evoked by CQ [104] and proenkephalin product, BAM8-22 [104,105,106]. Overexpression of TRPA1 in mast cells, keratinocytes, and dermal sensory neurons was found in human and murine AD models [103].

TRPA1 is an essential downstream mediator of GPCR signaling involved in histamine-independent itch [104]. Pruritogen-sensing GPCRs include the TSLP receptor, the bile acid receptor TGR5, and the MrgprA3 and MrgprC11, which can modulate TRPA1 positively [57,107,108]. In response to histaminergic signaling, TSLP released by keratinocytes activates TRPA1 downstream of the TSLP receptor and allows for calcium entry into the sensory neurons to promote itch signaling [107]. Similarly, a release of the pruritogenic neuropeptide GRP in response to the bile acids is diminished in pharmacological inhibition and genetic ablation of TRPA1 [108]. Thus, TRPA1 is activated and sensitized by TGR5 by a Gβγ- and PCK-dependent mechanism, and the overexpression of TGR5 that induces exacerbated spontaneous scratching is prevented with the treatment of a TRPA1 antagonist [108]. Histamine-independent itch mediators such as CQ and BAM8-22 peptide activate MrgprA3 with Gβγ signaling and MrgprC11 with PLC signaling, respectively [104]. One study, however, reported the lack of TRPA1 or TRPV1 involvement in the activation of GPCRs with CQ or histamine in inducing membrane depolarization and action potential at the peripheral C-fiber terminals of itch nerves [109]. Notably, this study found that, while the increase in intracellular calcium by CQ in nerve cell bodies dissociated from DRG is strictly dependent on TRPA1, TRPA1 is not required for the action potential elicited by CQ at the C-fiber nerve terminals [109]. These findings indicate that TRPA1 plays a distinct role at specific sites in the scratching response.

In addition to its relevance to GPCRs, TRPA1 has also been shown to interact with another ion channel, specifically the Nav1.7 channel. Methylglyoxal, an endogenous reactive carbonyl compound that plays a crucial role in the pathogenesis of diabetic neuropathy, has been found to activate both TRPA1 and Nav1.7 channels, leading to the induction of scratching behavior in response to methylglyoxal treatment [110]. Furthermore, the physiological role of extracellular microRNAs (miRNAs) as potential disease biomarkers has also been investigated in the context of itch signaling. Extracellular miR-711 has been shown to directly bind and activate TRPA1 in TRPA1-expressing heterologous cells and primary sensory neurons, resulting in scratching behavior in response to intradermal cheek injection, but not pain-related behavior. Interestingly, this effect was found to be independent of TLR7, a known modulator of itch sensation [111]. On the other hand, TLR7 agonist imiquimod (IQ) has been identified as another pruritogen via direct interaction with TRPA1, inducing itch-like behaviors in both zebrafish larvae and mice [112].

In TRPA1-deficient mice, scratching and general responses to AD seem diminished [113]. Additionally, both TRPA1 and TRPV1 channels are crucial for generating spontaneous scratching in a mouse ACD model [114]. A proinflammatory role for TRPA1 has been observed, evidenced by a suppressed cytokine response, epidermal thickening, and reductions in other features of inflammation [113]. Thus, the current understating of the pathology associated with TRPA1 in the skin requires further investigations. However, its involvement in pruriception, acting downstream of the GPCRs, makes it a potential drug target for alleviating the itch.

### 4.2. Sensory TRPV1 in Itch

TRPV1 is a subfamily of temperature-sensitive TRP channels activated by noxious temperatures greater than 43 °C, capsaicin, and low pH [115]. Furthermore, this channel can be directly and indirectly sensitized by various pro-inflammatory or pruritogenic agents, including histamine, proteases, sphingosine 1-phosphate (S1P), and IQ, which can endogenously mediate phosphorylation of intracellular domains by PKC, PKA, and other kinases [116,117]. The primary function of TRPV1 is the sensation of pain, with its expression mostly found in peripheral sensory nerves in the skin and central nerve endings in the DRG [118]. However, recent findings have reported TRPV1 distribution in other non-neuronal cells such as epidermal keratinocytes, T-cells, mast cells, leukocytes, macrophages, and sweat gland cells [119]. Therefore, when developing therapeutics for this channel, considering the impact on all populations of this channel distributed widely in various tissues and organs is essential.

TRPV1 is involved in histamine-dependent itch, which is facilitated by the co-expression of H1R signaling in sensory neurons [35], and histamine-induced scratching behaviors are abolished in TRPV1 knockout mice [36]. The molecular mechanism of the transduction of the histaminergic itch signal to evoke the scratching response has recently been discovered. It has been found that H1R directly binds to the deSUMOylated TRPV1 C terminus, facilitated by histamine treatment [120]. TRPV1 sensitization can also be induced by histamine-independent itch signaling, such as endogenous proteases that activate PAR-1 and PAR-4, which have been implicated in AD [121]. Thus, TRPV1 plays a critical role in itch signals associated with histamine-dependent and -independent itch.

IQ, an agonist of TLR7, also evokes itch-associated responses with the presence of TRPV1 [122]. Although research has found sex differences in TRPV1 and TRPA1 knockout mice regarding IQ-induced psoriatic dermatitis [123], these two channels may play a role in developing itch sensation. Lysophosphatidic acid (LPA) is another itch mediator associated with TRPV1 and TRPA1. Either TRPV1 or TRPA1 knockout mice show reduced LPA-induced DRG neuron activations and scratching responses after intradermal LPA administration [124]. Moreover, a low concentration of S1P evokes acute itch, whereas a high concentration causes pain and itch [125]. Studies have revealed that the co-expression of S1P receptor 3 (S1PR3) with TRPV1 leads to the induction of acute pain and heat hypersensitivity in response to S1P. However, when S1PR3 and TRPA1 are co-expressed, they mediate S1P-induced itch responses [120], suggesting that distinct mechanisms are involved in the signaling of itch and pain, and the interplay between TRPV1 and TRPA1 may play a role in the itch sensation.

Although the downstream signaling of histamine receptor subtypes H1R and H4R involves TRPV1, TRPA1 is also associated with histamine-induced pruritus transduction via H4R [126]. Periostin, a fasciclin extracellular matrix protein, potentially involves the activation of both TRPV1 and TRPA1 in the context of chronic allergic itch conditions [127]. This study revealed that periostin binds to the integrin αVβ3 in DRG sensory neurons, leading to the release of the neuropeptide NPPB due to TRPV1- and TRPA1-induced neuronal depolarization, which in turn contributes to the itch signal. In TRPV1 and TRPV4 knockout mice, cinnamaldehyde (CA)-induced scratching behavior was diminished, compared to wild-type mice, while TRPA1 knockout mice did not show the similar reduction in scratching behavior [128]. These findings suggest crosstalk between TRP channels and GPCRs, and the subsequent itch signaling transduction.

### 4.3. Sensory TRPV4 in Itch

TRPV4 is another temperature-sensitive TRP channel activated at 27 and 35 °C [88]. While TRPV4 is primarily expressed in keratinocytes, research has shown that the expression of TRPV4 in subsets of DRG sensory neurons is required for both CQ- and histamine-induced itch transmission [96]. In this study, the knockdown of TRPV4 in DRG neurons using intrathecal small interfering ribonucleic acid (siRNA) injection significantly reduced CQ or histamine-induced scratching behaviors compared to mice injected with negative control. Furthermore, the function of TRPV4 in complexes in DRG neurons and HEK293 cells depends on TRPV1, indicating that TRPV1 facilitates the TRPV4 responses to pruritogenic agents [96]. Thus, TRPV4 in sensory neurons plays a differential role in regulating itch signals.

### 4.4. Sensory TRPM8 in Itch

The activation of TRPM8 is induced by temperatures in the range of 8–28 °C and by natural compounds that can cause cooling sensations, such as menthone, menthol, and eucalyptol [129]. Cooling by applying cold water or ice has been commonly used to relieve itch, suggesting that TRPM8 activation can regulate itch transmission [130]. Moreover, cooling and menthol-induced TRPM8 activation can inhibit histamine-dependent and -independent itch pathways in neurons expressing TRPM8 [131]. Importantly, a TRPM8 agonist cooling compound, formed by combining with (1R,2S,5R)-N-(2-(2-pyridinyl)ethyl)-2-ispropyl-5-methylcyclohexancarboxamide and menthoxypropanediol, show a stronger activation than menthol [132]. The application of a lotion containing this compound ameliorated severe pruritus in a dry skin-associated chronic itch model, indicating that TRPM8 may play a role as an itch modulator. However, further research is required to identify a specific neural circuit and potential cellular mechanism for treating chronic itch.

### 4.5. Sensory TRPC3 and TRPC4 in Itch

TRPC channels are also calcium-permeable nonselective cation channels [133]. TRPC3 is particularly prevalent in DRG neurons, and its role in the histamine-independent itch pathway has been demonstrated through the reduction in scratching responses induced by TRPC3 agonist GSK1702934A, as well as endothelin-1 and SLIGRL in TRPC3 knockout mice [134], highlighting the role of TRPC3 in the histamine-independent itch pathway. Furthermore, research has demonstrated that TRPC3 is also expressed in MRGPRD-positive non-peptidergic C fiber nociceptors, where MRGPRD is a GPCR member D that mediates β-alanine-induced itch sensations [135]. However, the role of TRPC3 downstream of MRGPRD-positive neurons remains to be further investigated.

In DRG neurons, TRPC4 is co-expressed with serotonin receptor subtype 2B (HTR-2B) and mediates selective serotonin reuptake inhibitors (SSRIs)-evoked pruritus [136]. Sertraline, a commonly prescribed SSRI medication, elicited a robust itch response when administered subcutaneously. However, this response was significantly diminished in TRPC4 knockout mice without affecting the function of TRPA1 or TRPV1. Although TRPC4 antagonists, such as ML204, have been discovered [137], further research for its impact on itch responses is needed.

## 5. Antipruritic Compounds Targeting TRP Channels and Future Perspective

Several potential antipruritic compounds have been developed to target various TRP channels. Table 1 shows a list of published studies in which the effect of antipruritic compounds is attained through the modulation of TRP channels in diverse itch-related models. While some of these compounds are applied locally to the skin, others are delivered systemically. The expression of TRP channels is not limited to the skin or sensory neurons, as they are present in various tissues throughout the body. Therefore, systemically applied antipruritic compounds may have off-target effects on TRP channels in other physiological processes in non-itch-related tissues. For instance, the TRPV1 antagonist AMG-9810 was discontinued in the phase I clinical trial due to the side effect of hyperthermia, an abnormal rise in body temperature from a failure of the body’s heat-regulating mechanism [138]. Furthermore, the majority of antipruritic compounds have been investigated via restricted administration routes. Notably, some compounds (A-967079 and Resolvin D3) have demonstrated their efficacy through local and systemic administration (Figure 1).

As TRP channels have distinct functional roles in specific sites, global knockout studies may not provide tissue-specific information. Only studies on TRPV4 have demonstrated the effects of global or conditional knockout of TRPV4, including in skin keratinocytes, sensory neurons, and macrophages [19]. The use of global knockout studies to investigate the behavioral aspects of itch may be limited due to the dynamic nature of this process and the potential for the role of TRP channels to change over time. Therefore, to gain a more specific understanding of the tissue-specific role of TRP channels in itch, complementary approaches, such as tissue-specific knockouts and conditional knockouts, are considered.

## Figures and Tables

**Table 1:** Antipruritic compounds through TRP channels in various itch-related models.

Targets	Pharmaceuticals	Models	Route	Ref.
TRPA1	HC-030031 (antagonist)	DNCB-induced AD in mice	Intraperitoneal (100 mg/kg)	[139]
		Oxazolone-induced chronic dermatitis in mice	Intraperitoneal (60 mg/kg)	[113]
	A-967079 (antagonist)	Oxazolone-induced chronic dermatitis in mice	Intraperitoneal (100 mg/kg)	
		Tacrolimus-induced pruritus in chronic contact hypersensitivity mice	Topical (30 mg/kg)	[140]
TRPV1	Resolvin D3 (antagonist)	Imiquimod-induced spontaneous scratching and alloknesis in mice	Intraperitoneal (2.8 mg/kg) Intradermal (100 ng/100 µL) Intrathecal (10 ng/10 µL)	[141]
	PAC-14028 (antagonist)	Df extract-induced AD in mice	Oral (10–30 mg/kg)	[142]
		Oxazolone-induced chronic dermatitis in mice	Topical (50 µL of 1% cream)	[143]
		Mild to moderate AD in human	Topical (1% cream)	[144]
	Capsaicin (agonist)	Notalgia paraesthetica in human	Topical (8% patch)	[145]
		Brachioradial pruritus in human		[146]
	SB705498 (antagonist)	Histamine-induced pruritus in human	Topical (3% cream)	[147]
TRPV3	Citrusinine-II (antagonist)	AEW- and histamine-induced scratching in mice	Intradermal, transdermal (5–10 µM/50 µL)	[148]
		Histamine-induced pruritus in mice	Intradermal, transdermal (10 µM/50 µL)	
	Osthole (antagonist)	AEW- and histamine-induced scratching in mice	Intradermal (30–300 nM/50 µL)	[149]
		Histamine-induced pruritus in mice	Intradermal (300 nM/50 µL)	
	Forsythoside B (antagonist)	AEW- and histamine-induced scratching in mice	Intradermal (3–30 µM/50 µL)	[150]
		Histamine-induced pruritus in mice	Intradermal (0.3–30 µM/50 µL)	
		Carvacrol-induced pruritus in mice	Intradermal (30–300 µM/50 µL)	
	Verbascoside (antagonist)	Carvacrol-induced pruritus in mice	Intradermal (300 µM/50 µL)	[151]
	Isochlorogenic acid A (antagonist)	Carvacrol-induced pruritus in mice	Transdermal (1 mM/50 µL)	[152]
	Isochlorogenic acid B (antagonist)		Transdermal (1 mM/50 µL)	
	Scutellarein (antagonist)	Carvacrol-induced pruritus in mice	Subcutaneous (0.2–0.5 mg/kg)	[153]
		2,4-dinitrofluorobenzene-induceddermatitis and pruritus in mice	Subcutaneous (0.2–0.5 mg/kg)	
	Dyclonine (antagonist)	Carvacrol-induced pruritus in mice	Intradermal (10–50 µM/50 µL)	[154]
	Trpvicin (antagonist)	SLIGRL-induced pruritus in mice	Intradermal (10–100 µM/50 µL)	[155]
		Calcipotriol-induced pruritus in mice	Oral (100 mg/kg)	
TRPV4	Vitexin (antagonist)	Histamine-, C48/80-, chloroquine-, and BAM8-22-induced acute, and dry-skin-induced chronic itch in mice	Intravenous (7.5 mg/kg)	[156]
		AEW-induced dry skin causing chronic itch in mice	Intraperitoneal (7.5 mg/kg)	
	Cimifugin (antagonist)	GSK101-induced acute and Imiquimod-induced chronic itch in mice	Intragastric (75 mg/kg, 100 µL)	[157]

DNCB, 2,4-dinitrochlorbenzene; AD, atopic dermatitis; Df, dermatophagoides farina; NP, notalgia paraesthetica; BRP, brachioradial pruritus; AEW, acetone–ether–water; TRPV, transient receptor potential vanilloid.

## Data Availability

Data sharing is not applicable.
